# Multiparametric evaluation of a magnetic field source for thermomagnetic generator

**DOI:** 10.1016/j.isci.2026.117367

**Published:** 2026-08-27

**Authors:** Radel Gimaev, Andrej Kitanovski, Katja Klinar

**Affiliations:** 1 University of Ljubljana, Faculty of Mechanical Engineering, Chair of Thermal and Environmental Engineering, Laboratory for Refrigeration and District Energy, Askerceva 6, 1000 Ljubljana, Slovenia

**Keywords:** thermomagnetic, magnetic field source, waste heat, magnetic energy conversion, heat transfer

## Abstract

Thermomagnetic generators (TMGs) represent a promising solid-state technology for low-grade waste heat recovery. While extensive research has addressed working materials and heat transfer optimization, systematic analysis of magnetic field source design in TMGs remains underexplored. This study presents a parametric finite element analysis of the magnetic field source in a TMG. Magnetostatic and transient simulations were performed to evaluate the effects of permanent magnet (PM) material (neodymium-iron-boron [NdFeB], Alnico, and Ferrite), its dimensions, and air gap width, on the TMG output power and specific power. It is shown that reducing PM thickness yields a substantial gain in specific power output per PM mass, while an increased air gap can be maintained without significant effects on output power. The study also investigates Alnico PM as a sustainable, rare-earth-free alternative. These findings contribute to the development of mass-optimized and cost-effective thermomagnetic energy harvesters.

## Highlights

• Parametric study of magnetic field source design for thermomagnetic generators
• A 4-fold permanent magnet thickness reduction yields 2.7× higher specific power
• Doubling the air gap from 4 to 8 mm maintains or slightly improves the output power
• Alnico at 9 mm thickness matches NdFeB specific power without rare earths

## Graphical abstract

**Figure:**
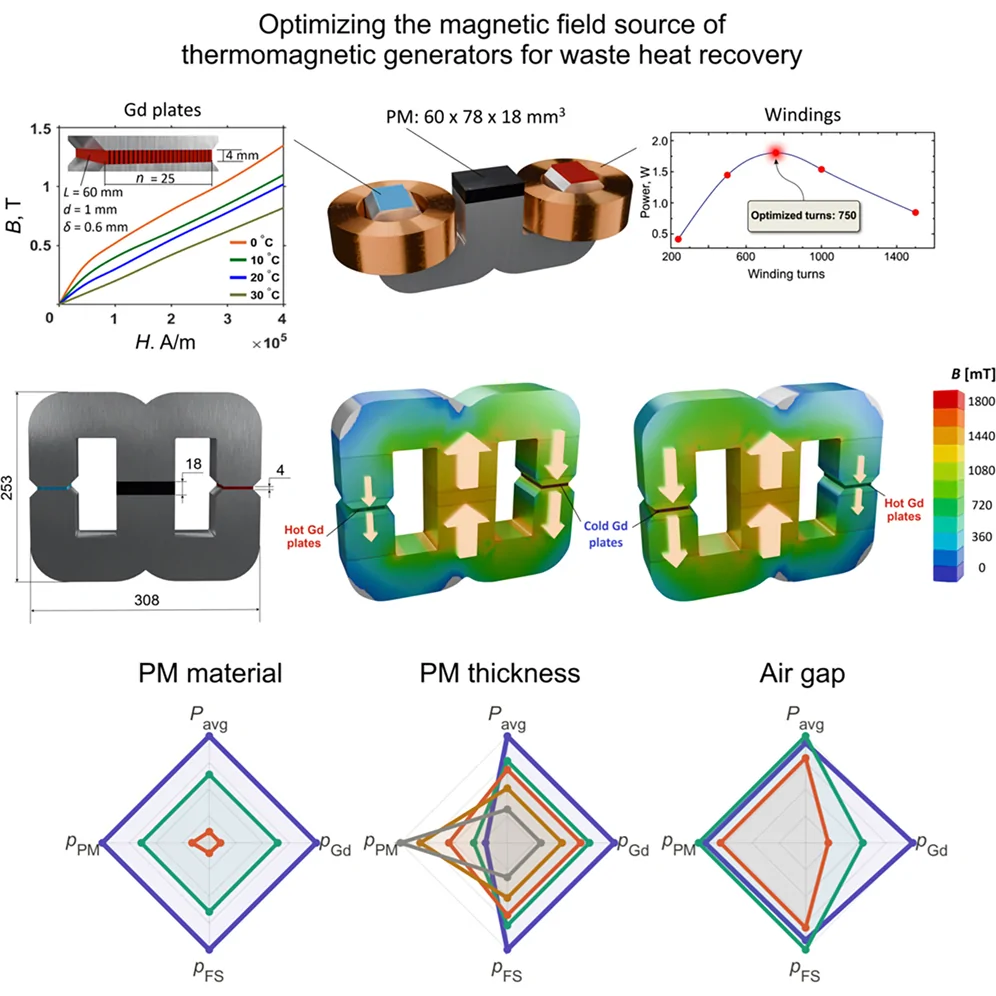

## Teaser

physics; thermodynamics; heat transfer; applied sciences

## Introduction

All energy conversion processes inevitably generate waste heat. Globally, this equates to 50% of the total 100% energy input consumed, positioning waste heat as one of the world’s largest untapped resources.^1^ Principal waste heat sources span industrial processes, power generation and distribution, the building sector, and transportation.^2^ Classification schemes for waste heat vary across studies due to differing temperature thresholds and applications; for this analysis, we adopt the categories defined by the EU Joint Research Centre^3^: low-grade (40°C–99°C), low-to-medium grade (100°C–149°C), medium grade (150°C–500°C), and high-grade (>500°C).

Renewable heat constitutes another vast untapped resource.^4^ Accordingly, integrating waste and renewable heat sources offers the optimal pathway toward resilient, sustainable heating, and cooling systems. Both waste and renewable heat can be harnessed or upgraded for diverse heating and cooling applications, including district heating,^5^ district and process cooling,^6^ and a broad array of industrial processes.^7^^,^^8^ Renewable or waste heat from low-to-medium grade and higher (i.e., above 100°C) categories lends itself particularly well to power generation via established technologies^9^ such as organic Rankine cycles (ORC)^10^ or Kalina cycles.^11^ These can serve for electricity production with heat sources between 80°C and 350°C.^12^^,^^13^^,^^14^ For higher temperature levels, steam, gas and combined turbine cycles are very well developed and broadly applied today.^15^^,^^16^^,^^17^ A distinct domain encompasses gas-cycle linear generators, such as mechanical Stirling^18^^,^^19^ or thermoacoustic devices.^20^^,^^21^ These generators enable operation across smaller temperature differentials between heat source and sink, while accommodating a broad spectrum of conditions: from low to very high temperatures.^22^^,^^23^

From a heat-to-power perspective, categories ≥100°C are relatively well-served by established conventional and emerging technologies. However, the conversion of heat to power at low temperatures below 80°C represents a limit for all aforementioned technologies. Low-temperature heat sources exhibit low exergy potential due to their proximity to ambient temperatures, making them highly susceptible to inefficiencies from even small losses. On the other hand, low-grade waste heat accounts for up to 65% of all waste heat and represents the most untapped potential.^1^^,^^2^^,^^24^ Technologies are thus needed not only to reuse or upgrade it via heat pumps but also to convert it into valuable exergy, such as electricity. Dispersed sources of such waste heat can enable remote power generation where grid independence is essential or facilitate the transformation of vast thermal quantities into electricity, thereby mitigating environmental thermal pollution.

Thus, it is no mere coincidence that the global market for waste heat recovery harbors vast potential, with numerous studies projecting a compound annual growth rate (CAGR) of 7%–10% over the next five years to propel its value beyond $110 billion by 2031.^25^^,^^26^^,^^27^^,^^28^ Amid this landscape, the global waste-heat-to-power segment is projected to grow at 10% annually, attaining roughly $60 billion by 2031,^29^ while the European Union currently commands the worldwide market for waste heat recovery and conversion technologies.

This explains why researchers are increasingly focusing on solid-state technologies among which thermoelectrics are the most advanced and widely adopted.^30^^,^^31^ However, other solid-state thermal energy harvesting methods^32^^,^^33^—primarily pyroelectrics,^34^ thermomechanics,^35^ and thermomagnetics^32^^,^^36^—are also under active exploration. These emerging fields promise substantially higher energy efficiency for heat-to-power conversion compared with thermoelectrics, owing to material transitions and thermodynamic cycles that enable theoretically reversible processes.

Research on thermomagnetic devices published over the last five years demonstrates a range of scalable systems embodying diverse thermally activated principles, including pumping mechanisms,^37^^,^^38^^,^^39^ actuators and switches,^40^ power generators,^41^^,^^42^^,^^43^^,^^44^ and motors.^45^^,^^46^ These operate across varied conditions, including some unconventional ones.^47^ Parametric studies of PM configurations have been reported in the context of electric machines, including flux-switching machines^48^ and hybrid rare-earth/Ferrite PM motors aimed at reducing rare-earth content.^49^ While extensive efforts have addressed heat transfer optimization,^50^^,^^51^ conceptual design,^52^ and materials,^53^^,^^54^^,^^55^ the research community lacks comprehensive studies on the performance of diverse magnetic field sources—their materials and dynamic or quasi-static operational characteristics.

In this work, we evaluate a static electro-permanent magnet (EPM)-based magnetic field source—originally developed, constructed, and tested for magnetocaloric cooling and heat pumping^56^—across various PM materials, thicknesses, and air gap configurations. This analysis targets dynamic performance and provides tailored guidelines for advancing thermomagnetic generator research. We contend that the magnetic field source—its structure, topography, and dynamic operation—remains a critical, underexplored area requiring thorough investigation to yield optimized solutions, especially as working material designs and materials have already been extensively studied in magnetocalorics.^57^

## Results

### Reference design and study scope

**Figure 1:**
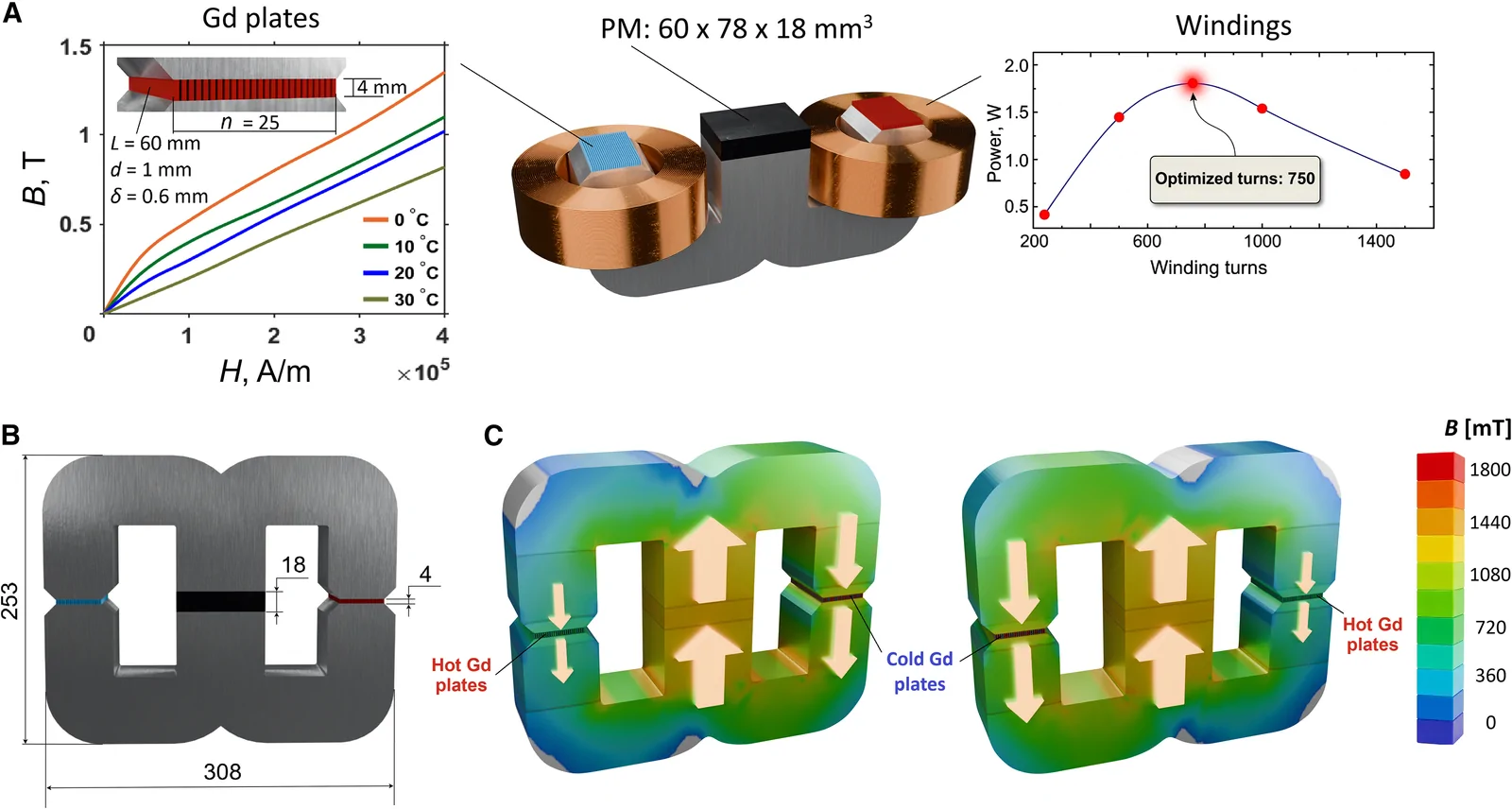
Magnetic system configuration and magnetic flux distribution (A) 3D assembly view showing the PM, windings, and layered Gd-plates. Left inset: *B*-*H* curves of Gd at different temperatures (0°C–30°C); *L* is plate length, *d* is plate thickness, *δ* is inter-plate gap, and *n* is number of plates. Right inset: TMG output power as a function of the number of turns per winding, with an optimal value of 750 turns. (B) Magnetic flux distribution (arrows) for the thermal state with left Gd assembly at 0°C, right Gd assembly at 30°C; size unit is mm. (C) FEM simulation of the magnetic flux density *B* distribution for two distinct thermal states: (left) left Gd assembly at 0°C, right Gd assembly at 30°C and (right) left Gd assembly at 30°C, right Gd assembly at 0°C.

**Figure 2:**
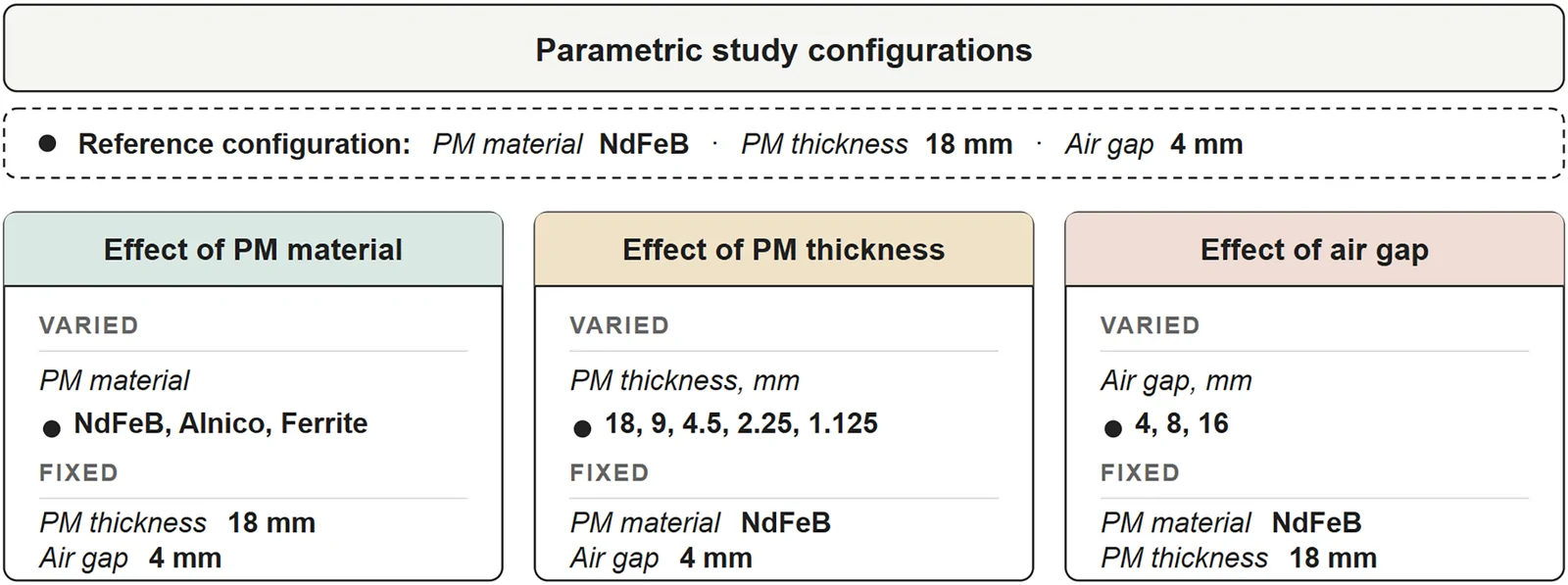
Magnetic field source design configurations studied in this work

The electro-permanent magnet field source used as the reference design is shown in Figure 1, with key dimensions and the layered gadolinium (Gd) working material assembly indicated. The PM materials investigated and their key magnetic properties are listed in Table 1; the full set of parametric configurations (PM material, thickness, and air gap) is summarized in Figure 2; the output parameters extracted from the simulations and their definitions are given in Table 2. Full methodological details, including model validation and simulation setup, are provided in STAR Methods.

**Table 1:** Magnetic properties of PM materials investigated in this study

Material	*B*_r_ (mT)	*H*_c_ (kA/m)	(*BH*)_max_ (kJ/m^3^)	References
NdFeB N45	1,370	945	358	^58^
Alnico 9	1,120	109	83.6	^59^
Ferrite Y30	400	175	30	Eclipse Magnetics^60^

**Table 2:** Output parameters extracted from magnetostatic and transient simulations

Output parameter	Evaluation method	Magnetostatic	Transient
*Φ*_L_, *Φ*_R_	Fields calculator	✓	–
Δ*Φ*	|Φ_*L*_-Φ_*R*_|	✓	–
Δ*B*_int_	Fields calculator	✓	–
*P*(t)	Maxwell-Twin Builder	–	✓
*I*(t)	Maxwell-Twin Builder	–	✓
*U*(t)	Maxwell-Twin Builder	–	✓
*P*_avg_	$\frac{1}{t_{cycle}}\int_{0}^{t_{cycle}}P\left(t\right)dt$	–	✓
*p*_PM_	*P*_*avg*_/*m*_*PM*_	–	✓
*p*_FS_	${P_{avg}/m}_{FS}$	–	✓
*p*_Gd_	*P*_*avg*_/*m*_*Gd*_	–	✓

### Magnetostatic analysis

**Figure 3:**
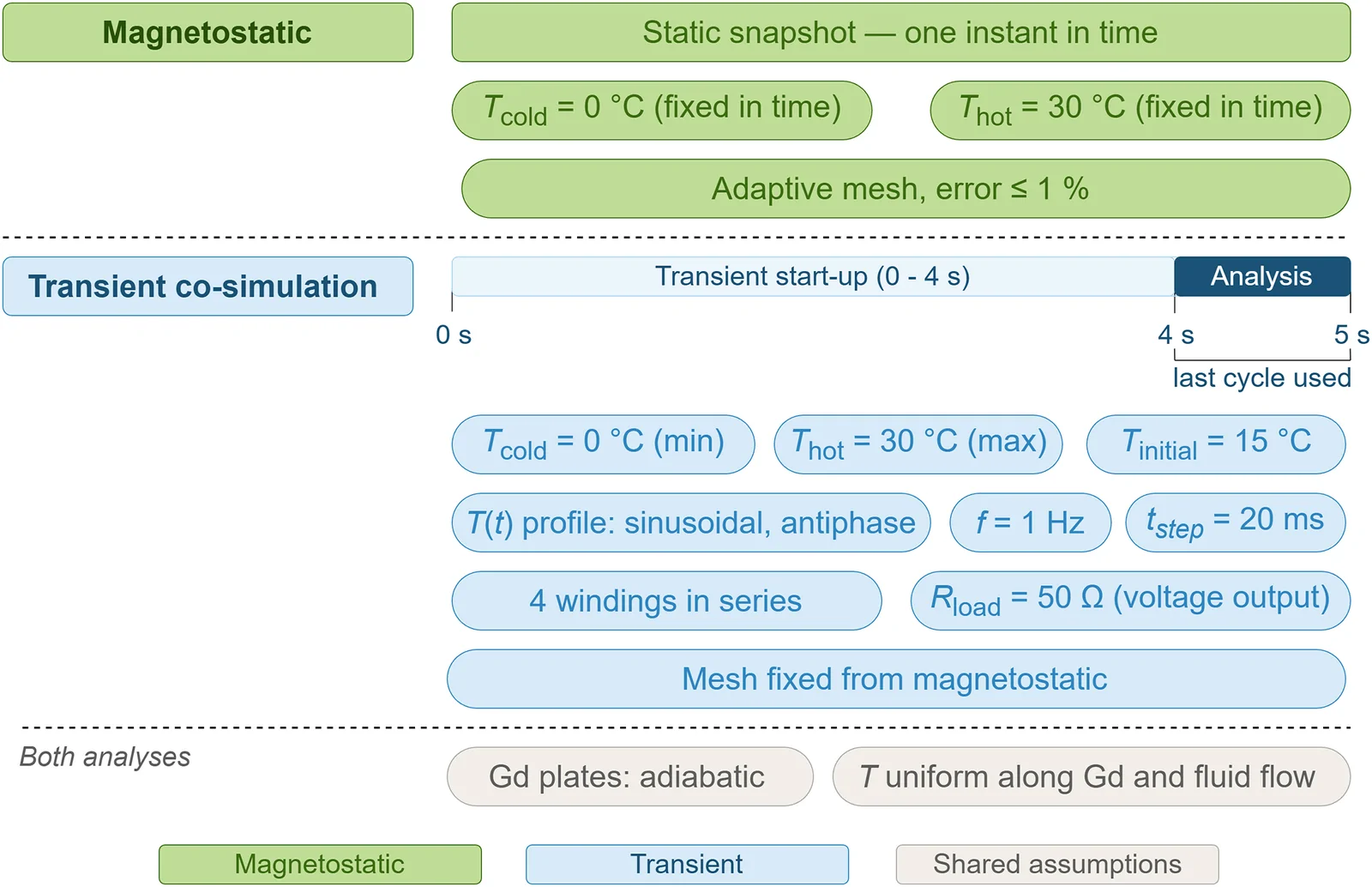
Simulation conditions for magnetostatic and transient analyses

**Figure 4:**
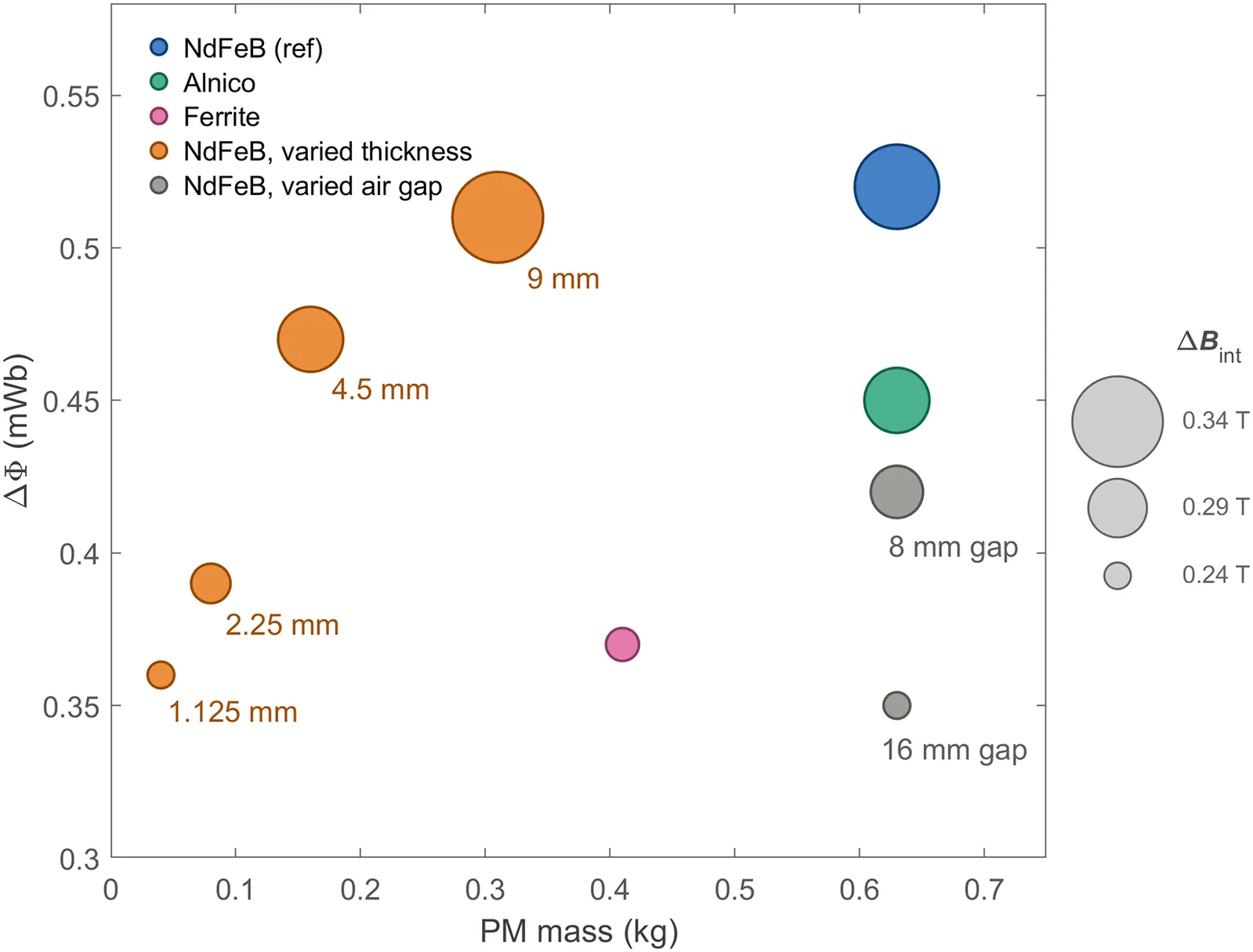
Magnetic flux change Δ*Φ* as a function of PM mass for studied configurations Bubble size represents the internal flux density change Δ*B*_int_. Alnico and Ferrite are shown at reference geometry only (18 and 4 mm gap); PM thickness and air gap variations were performed for NdFeB only.

The magnetostatic analysis (conditions given in the Figure 3) of the reference design (Figure 1) yielded a magnetic flux density change inside the Gd of Δ*B*_*int*_ = 0.33 T and a magnetic flux change through the windings Δ*Φ* = 0.52 mWb. The results for all configurations are presented in Figure 4 and summarized in Table S3.

The comparative analysis reveals that NdFeB provides a Δ*Φ* approximately 15% and 40% higher than Alnico and Ferrite, respectively, at identical geometry (18 mm PM thickness, 4 mm air gap; Figure 4, blue, green, and pink circles; Table S3). Notably, Alnico is rare-earth free and retains approximately 87% of the NdFeB flux change at the same geometry (Figure 4, green vs. blue circle; Table S3), making it a competitive alternative where the thermal stability and absence of rare-earth metals are prioritized. Ferrite PM, despite providing a Δ*Φ* approximately 40% lower than NdFeB (Figure 4, pink vs. blue circle) at identical geometry, has a significantly lower mass density (5 vs. 7.5 g/cm^3^), resulting in a magnet mass of 0.41 kg compared with 0.63 kg for NdFeB (Table S3). This yields a higher Δ*Φ* per PM mass (0.90 vs. 0.83 mWb/kg), making Ferrite a viable option for applications where reduced PM mass and lower material cost are prioritized over absolute flux performance.

Reducing the thickness of NdFeB from 18 to 9 mm reduces Δ*Φ* by less than 2%, while halving the magnet mass (Figure 4, two uppermost orange circles), suggesting near-saturation of magnetic induction within the Gd plates at 9 mm. For thinner NdFeB PMs (1.125–2.25 mm), Δ*Φ* drops by 30%–35% (Figure 4, two lower-left orange circles). Increasing the air gap from 4 to 16 mm (NdFeB, 18 mm thickness) reduces Δ*Φ* by approximately 30% (Figure 4, gray circles), which is associated with an increase in the magnetic reluctance of the gap.

Based on magnetostatic analysis, the most promising configurations are NdFeB with 9 and 4.5 mm thickness (Figure 4, large orange circles—high Δ*Φ* at reduced PM mass); NdFeB with 8 mm air gap (Figure 4, gray circle—acceptable Δ*Φ* with increased manufacturing flexibility); and Alnico at reference geometry (Figure 4, green circle—comparative Δ*Φ* as rare-earth-free alternative).

### Transient analysis

**Figure 5:**
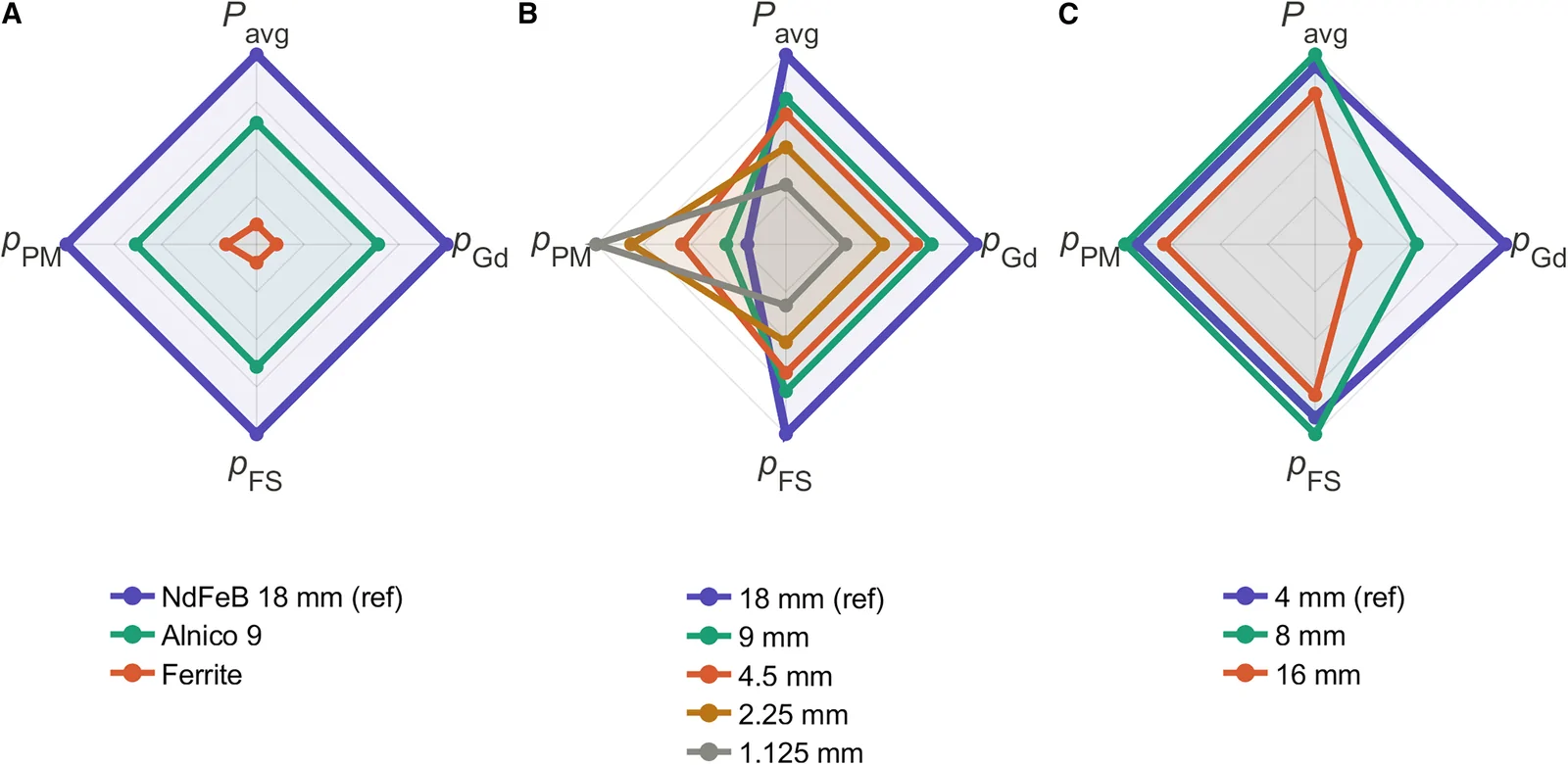
Comparison of TMG field source configurations in terms of *P*_avg_, *p*_PM_, *p*_FS_, and *p*_Gd_ (each axis normalized to its maximum value among the configurations shown in the respective panel) (A) Effect of PM material (18 mm PM thickness, 4 mm air gap); (B) effect of NdFeB PM thickness (4 mm air gap); (C) effect of air gap width (NdFeB, 18 mm PM thickness). Other PM dimensions are fixed at 60 × 78 mm (Figure 1). Simulation conditions are listed in Figure 3. Absolute values for all configurations are provided in Tables S4–S6.

The normalized performance comparison across all parametric configurations is presented in Figure 5, which summarizes the transient simulation results for variations in PM material (Figure 5A), PM thickness (Figure 5B), and air gap width (Figure 5C). In each panel, four performance metrics are shown: cycle-averaged output power *P*_avg_ and specific power per PM mass (*p*_PM_), field source mass (*p*_FS_), and Gd mass (*p*_Gd_). For each metric axis, the values are normalized to the maximum value among all configurations shown in that panel. For example, in Figure 5B, the 1.125 mm configuration has the highest *p*_PM_ (6.75 W/kg; Table S5) and therefore reaches the outer boundary on the *p*_PM_ axis, while the ref. 18 mm configuration (1.37 W/kg; Table S5) extends only to approximately one-fifth of that distance on the same axis. The detailed results for each configuration are provided in Tables S4–S6.

### Reference design

**Figure 6:**
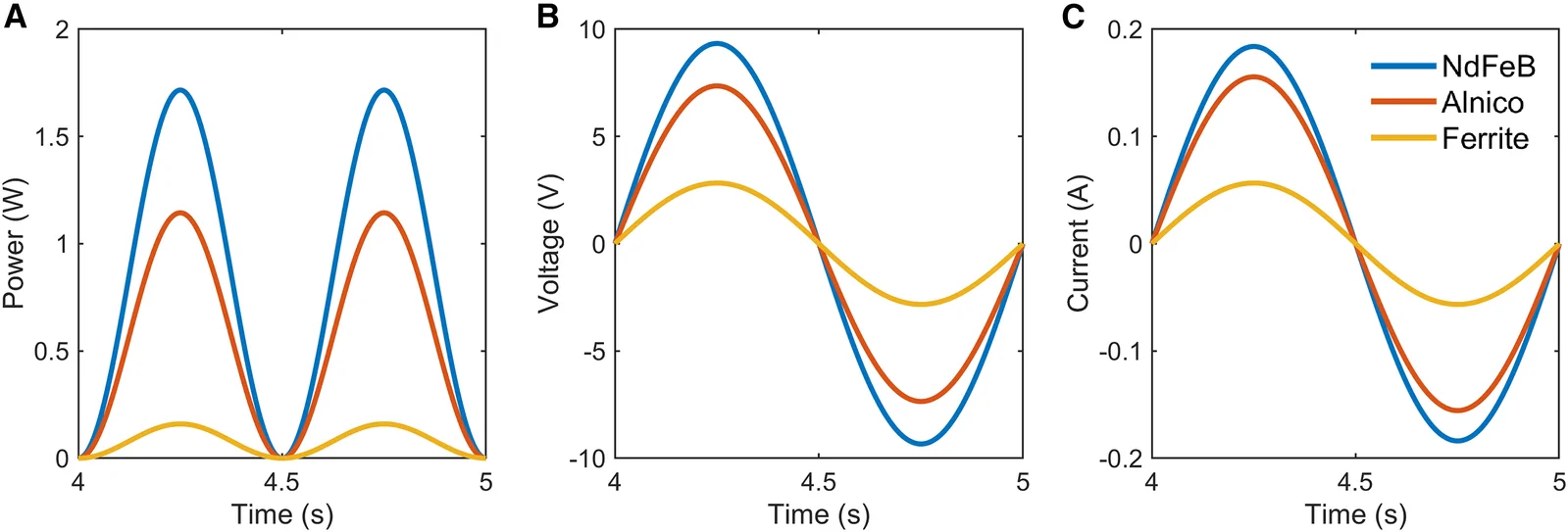
Electrical Output Characteristics of TMG Configurations with Different Permanent Magnets Time dependences of output power (A), voltage (B), and current (C) for TMG configurations with NdFeB, Alnico, and Ferrite PMs over the last simulated cycle (*t* = 4–5 s).

Transient simulations of the reference NdFeB design (Figure 1, 60 × 78 × 18 mm, 4 mm air gap) demonstrated an amplitude power output of 1.71 W with corresponding voltage and current amplitudes of 9.3 V and 0.19 A, respectively. The time dependencies of these parameters over several thermomagnetic generator (TMG) operating cycles are shown in Figure 6 (blue lines). The average power per cycle for the reference design was calculated to be *P*_avg_ = 0.86 W. This value corresponds to a specific power *p*_FS_ = 0.031 W/kg and *p*_Gd_ = 9.05 W/kg.

Numerical modeling was performed to optimize the field source design, with particular attention to the influence of PM material, PM thickness, and the air gap width on the TMG output power and specific power.

### Effect of PM type

Figure 6 shows the time dependences of the induced power, voltage, and current generated by the TMG using NdFeB, Alnico, and Ferrite PMs at reference geometry (60× 78 × 18 mm, 4 mm air gap).

The amplitude values of the power are 1.71, 1.1, and 0.17 W for the TMG based on NdFeB, Alnico, and Ferrite magnets, respectively. The corresponding voltage amplitudes are 9.3, 7.4, and 2.8 V, and the current amplitudes are 0.18, 0.15, and 0.06 A. The output performance metrics are summarized in Table S4; a normalized performance comparison is presented in Figure 5A.

The transient simulation results indicate that the NdFeB-based design yields the highest output power (*P*_avg_ = 0.86 W) and specific power (*p*_PM_ = 1.37 W/kg, *p*_FS_ = 0.031 W/kg, and *p*_Gd_ = 9.05 W/kg) among the three PM materials studied. The 36% decrease in power observed for Alnico compared with NdFeB is attributed to its lower maximum energy product (*BH*)_max_. However, Alnico remains a viable compromise for TMG applications where high thermal stability and absence of rare-earth metals are prioritized.

Ferrite magnets demonstrated the lowest output power and specific power approximately one order of magnitude lower than the NdFeB-based reference design. While Ferrite magnets are characterized by low cost and high corrosion resistance, their use results in significantly lower output power in this TMG geometry.

### Effect of reducing the thickness of the PM

Numerical simulations were performed using NdFeB PM with thicknesses of 9, 4.5, 2.25, and 1.125 mm, corresponding to a reduction in the PM mass from the reference design by factors of 2, 4, 8, and 16, respectively. The calculated performance parameters are summarized in Table S5. A normalized performance comparison is presented in Figure 5B.

The simulations show that the TMG output power decreases more slowly than the magnet mass. Reducing the magnet thickness (and mass) by a factor of 16 (from 18 to 1.125 mm) decreases the *P*_avg_ by only 3.2 times, while *p*_PM_ increases from 1.37 to 6.75 W/kg, demonstrating more efficient material utilization.

Reducing the thickness by a factor of 4 (from 18 to 4.5 mm) represents the most effective compromise: *P*_avg_ decreases by less than 1.5 times (from 0.86 to 0.59 W), while *p*_PM_ increases 2.7-fold, from 1.37 to 3.69 W/kg.

### Effect of increasing the air gap

Numerical simulations were performed at gaps of 4, 8, and 16 mm. The calculated performance parameters are summarized in Table S6. A normalized performance comparison is presented in Figure 5C.

The Gd plates fill the entire air gap height, so increasing the gap also increases the Gd mass. This explains the decrease in *p*_Gd_ with increasing gap size.

The static magnetostatic results show that both the internal flux density change and the flux change through the windings decrease monotonically with increasing air gap (Δ*B*_int_ from 0.33 to 0.28 T and Δ*Φ* from 0.52 to 0.42 mWb between 4 and 8 mm; Table S3; Figure 4), as expected from the increasing magnetic reluctance of the gap. The cycle-averaged power, however, does not follow this static trend: increasing the air gap from 4 to 8 mm causes *P*_avg_ to rise slightly, from 0.86 to 0.92 W. The output voltage is set not only by the amplitude of the flux change but by its rate dΦ/dt throughout the sinusoidal temperature cycle. Because the magnetization of Gd depends nonlinearly on temperature near its transition, *Φ*(*t*) is non-sinusoidal even though *T*(*t*) is sinusoidal, so the induced voltage depends on the temperature sensitivity d*Φ*/d*T* at the instantaneous operating point and not on the static flux change alone. Increasing the air gap lowers the average field in the Gd and shifts its operating point toward the part of the *B*-*H* family where *Φ* varies most steeply with temperature near the transition; this steep variation is expected to occur closer to the mid-cycle temperature (*T* = 15°C), at which |d*T*/d*t*| is largest, so that the peak d*Φ*/dt is enhanced. This dynamic enhancement offsets the modest reduction in the static flux change, so *P*_avg_ remains nearly constant and even rises slightly, and is corroborated by the small increase in effective voltage (from 6.6 to 6.8 V; Table S6).

Increasing the gap further to 16 mm reduces *P*_avg_ to 0.73 W: the static flux change drops substantially, and the steep region of *Φ*(*T*) no longer coincides with the mid-cycle temperature, so both effects now act to reduce the output. The decrease in *p*_Gd_ with gap size is additionally driven by the larger Gd mass. The obtained results indicate that an air gap of 8 mm provides the optimal balance between TMG output performance and technological flexibility of the field source.

The simulations were performed at 1 Hz; within the low-frequency regime where eddy-current losses are negligible, and field penetration is complete, the relative ranking of configurations is expected to remain qualitatively similar at other operating frequencies.

## Discussion

**Figure 7:**
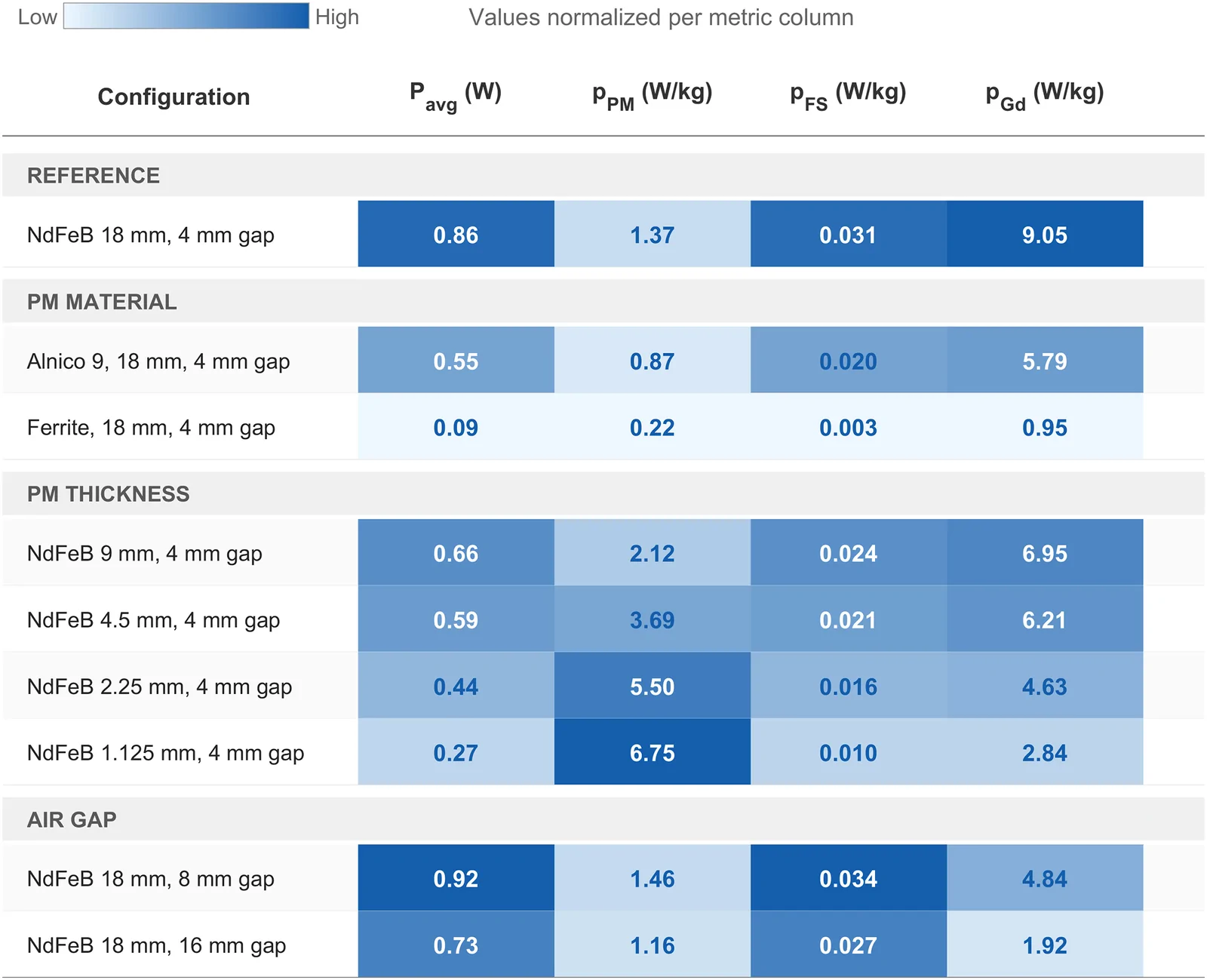
Comparative summary of TMG parametric analysis results across all studied configurations: *P*_avg_, *p*_PM_, *p*_FS_, and *p*_Gd_ Values are normalized independently per metric column to the maximum value among all configurations.

Building on the parametric results presented earlier, this section summarizes the key trends and their practical implications for TMG field source design. The parametric analysis results are summarized in Figure 7, which compares *P*_avg_, *p*_PM_, *p*_FS_, and *p*_Gd_ across all studied configurations, including variations in PM type, PM thickness, and air gap.

The highest *P*_avg_ is expectedly achieved with NdFeB magnets, attributed to their high maximum energy product (*BH*)_max_ resulting from the combination of high residual induction *B*_r_ and coercivity *H*_c_. Alnico magnets provide approximately 64% of the NdFeB power output but offer important advantages: absence of rare-earth metals and high thermal stability.

Reducing the magnet thickness decreases *P*_avg_ but significantly increases *p*_PM_, which is the key criterion for optimizing the PM material utilization. The most effective compromise is a magnet thickness of 4.5 or 9 mm, depending on the magnet material.

Increasing the air gap to 8 mm yields a slight improvement in *P*_avg_ (from 0.86 to 0.92 W) while providing greater design freedom for the TMG assembly and development. Further increase to 16 mm leads to performance deterioration due to field weakening and increased Gd mass.

The parametric variation of PM thickness and air gap was performed for NdFeB only (Figure 2). For Alnico, a 2-fold thickness reduction to 9 mm was selected as the candidate configuration, rather than the 4-fold reduction identified as optimal for NdFeB, since Alnico’s lower maximum energy product (*BH*)_max_ and coercivity are expected to make its output more sensitive to significant thickness reduction. The air gap values of 4 and 8 mm were carried over from the NdFeB parametric results as the most favorable configurations. Dedicated transient simulations were then performed for these selected combinations.

Based on comprehensive analysis, the following configurations are identified as optimal.

• NdFeB magnet with 4.5 mm thickness, 4 and 8 mm air gap;
• Alnico magnet with 9 mm thickness, 4 and 8 mm air gap.

**Figure 8:**
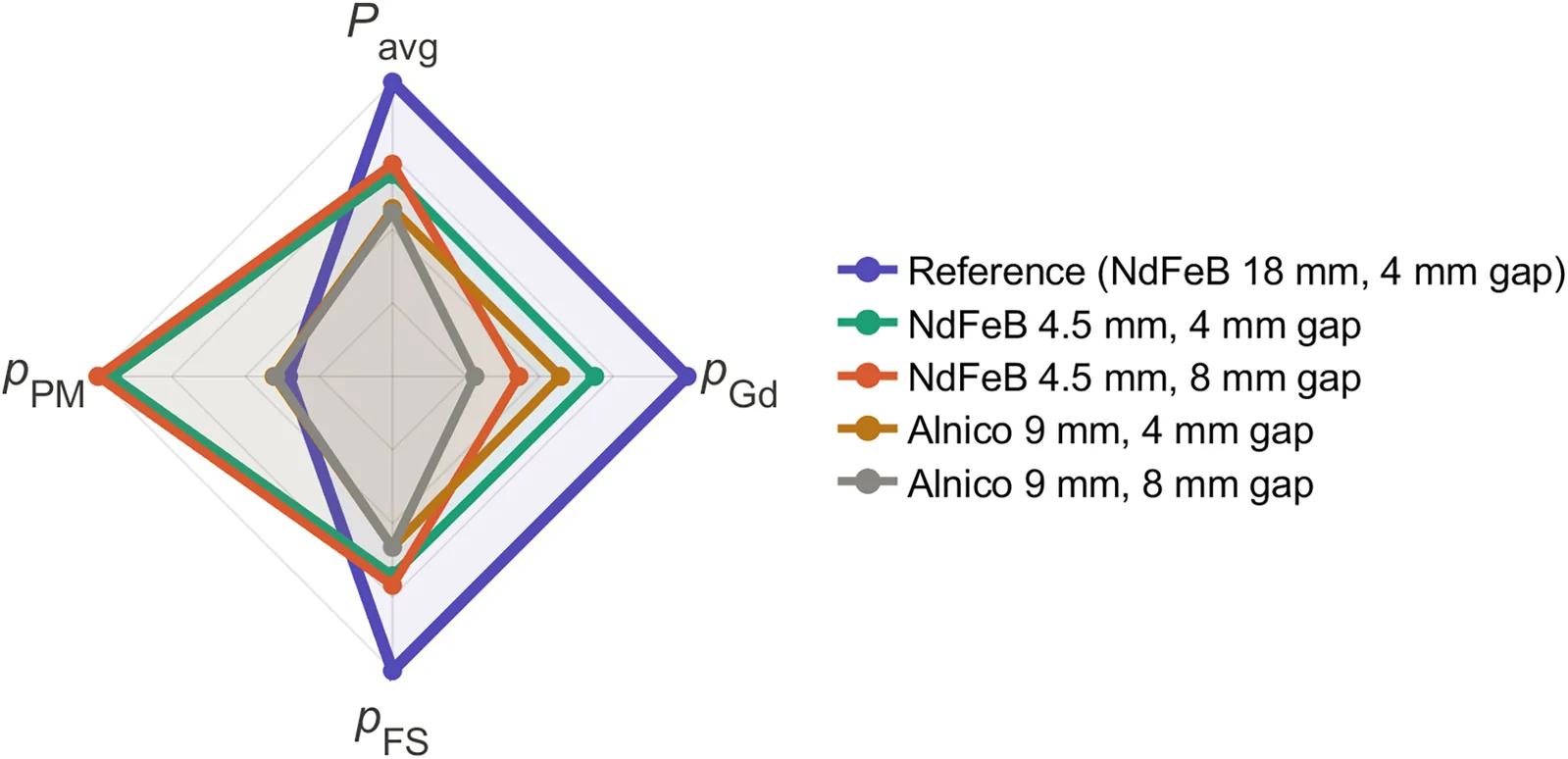
Comparison of the proposed TMG field source configurations relative to the reference design in terms of *P*_avg_, *p*_PM_, *p*_FS_, and *p*_Gd_ Each axis normalized to its maximum value among the configurations.

The results are presented in Table 3 and Figure 8. Replacing the 18 mm NdFeB with a 4.5 mm magnet reduces magnet mass by a factor of 4, while *P*_avg_ decreases by only 30% (from 0.86 to 0.59 W), resulting in a 2.7-fold increase in *p*_PM_ from 1.37 to 3.69 W/kg. Increasing the air gap to 8 mm causes a slight further drop in *P*_avg_ (to 0.62 W) while maintaining high *p*_PM_ (3.88 W/kg) and improving manufacturing flexibility.

**Table 3:** Output characteristics of the TMG for several proposed field source configurations, obtained from transient simulations (simulation conditions given in Figure 3)

Parameter	Reference design	NdFeB, 4.5 mm, 4 mm gap	NdFeB, 4.5 mm, 8 mm gap	Alnico, 9 mm, 4 mm gap	Alnico, 9 mm, 8 mm gap
PM mass (kg)	0.63	0.16	0.16	0.31	0.31
Yoke mass (kg)	26.8	27.3	26.9	27.1	26.8
Gd mass (kg)	0.095	0.095	0.19	0.095	0.19
*U*_*eff*_ (V)	6.6	5.4	5.5	4.9	4.88
*I*_*eff*_ (A)	0.13	0.11	0.11	0.1	0.1
*P*_*avg*_ (W)	0.86	0.59	0.62	0.49	0.48
*p*_*PM*_ (W/kg)	1.37	3.69	3.88	1.56	1.54
*p*_*FS*_ (W/kg)	0.031	0.021	0.022	0.018	0.018
*p*_*Gd*_ (W/kg)	9.05	6.21	3.88	5.16	2.53

Alnico configurations, despite lower *P*_avg_ (0.48–0.49 W), offer a technologically robust alternative with *p*_PM_ comparable with the reference NdFeB design (1.54–1.56 W/kg). However, considering the significant reduction in the mass of the magnetic system compared with the reference NdFeB design, the Alnico configurations with 9 mm PM thickness represent the most effective balance between specific power and output performance.

The present findings are based on a single EPM-based TMG configuration with Gd as the working material at 1 Hz; the applicability of the identified trends to other TMG architectures, working materials, or operating conditions may require dedicated investigation.

This study presents a parametric FEM analysis of the magnetic field source in a TMG, evaluating the effects of PM material, thickness, and air gap on output power and specific power.

The key findings of the study are the following.

1. Reducing the NdFeB PM thickness provides the most effective pathway for optimizing magnet material utilization. A 4-fold reduction in PM thickness results in a 2.7-fold increase in specific TMG power per PM mass, while absolute output power decreases by less than 1.5 times. Even a 16-fold reduction in PM mass decreases absolute output power by only 3.2 times.
2. For the reference configuration (NdFeB, 18 mm PM thickness, 4 mm air gap), the air gap of the magnetic field source can be doubled without compromising TMG output performance. Absolute output power remains essentially unchanged, while greater design freedom is gained for the TMG assembly. Further increase of the air gap leads to output power deterioration due to reduced magnetic flux change through the windings and increased Gd mass.
3. Among the studied PM materials, NdFeB delivers the highest absolute output power (0.86 W) at the reference geometry. Alnico provides approximately 64% of the NdFeB output, while Ferrite yields nearly an order of magnitude lower power.
4. Alnico represents a viable rare-earth-free alternative to NdFeB. With reduced PM thickness (9 mm), it achieves specific output power per PM mass comparable with the reference NdFeB design.
5. Based on a comprehensive parametric analysis, several optimal magnetic field source configurations are identified: 4.5 mm NdFeB and 9 mm Alnico, both with a 4–8 mm air gap.

Based on the key findings and our observations, we provide here some basic guidelines for the future research of TMG devices.

1. Rare-earth-free magnetic field source designs, particularly those based on Alnico, deserve dedicated research attention. Future studies should explore Alnico-specific geometries rather than simply substituting Alnico into layouts originally optimized for NdFeB, since the differing *B*-*H* characteristics and lower coercivity of Alnico may favor distinct topological solutions.
2. Parametric analysis of magnetic field sources should be extended to alternative static topologies beyond the EPM configuration examined here. Halbach arrays, flux-concentrating pole-piece designs, and multi-pole or segmented PM topologies represent promising candidates whose performance under realistic TMG operating conditions remains underexplored.
3. The present work focused on optimizing the magnetic field source for a fixed working material (Gd). Whether the identified optimal configurations remain optimal for other magnetic materials (with different Curie temperatures and *B*-*H* characteristics near the phase transition) remains an open question. Joint optimization of the magnetic field source and the working material represents a natural direction for further research.
4. Experimental validation of the complete TMG system, including the Gd working material and induction windings under realistic operating conditions, represents a critical next step and an important direction for future work.
5. Hybrid PM configurations combining NdFeB with low-cost Ferrite represent a promising direction for future investigation and could reveal performance-cost trade-offs complementary to the single-material analysis presented here.

### Limitations of the study

This study is based on a single EPM-based TMG configuration with gadolinium at 1 Hz, and the results are best interpreted as comparative performance indicators rather than absolute predictions for a specific system. The parametric variation of PM thickness and air gap was performed for NdFeB only; Alnico configurations were selected by physical reasoning. Heat transfer is not modeled, and working material temperatures are prescribed simulation inputs. Experimental validation of the complete TMG system remains an important direction for future work; the magnetic field source alone has been validated in prior studies.^56^^,^^61^

## Resource availability

### Lead contact

Requests for further information and resources should be directed to and will be fulfilled by the lead contact, Andrej Kitanovski (andrej.kitanovski@fs.uni-lj.si).

### Materials availability

This study did not generate new materials.

### Data and code availability

The simulation data supporting the findings of this study are available from the corresponding author upon request. This paper does not report original code. Any additional information required to reanalyze the data reported in this paper is available from the lead contact upon request.

## Acknowledgments

The authors acknowledge the financial support of the Slovenian Research and Innovation Agency for the research core funding no. P2-0223 Heat and Mass Transfer.

## Author contributions

R.G., methodology, software, validation, investigation, writing – original draft, writing – review and editing, and visualization; A.K., conceptualization, validation, investigation, writing – original draft, writing – review and editing, visualization, and funding acquisition; K.K., conceptualization, validation, writing – original draft, writing – review and editing, and visualization.

## Declaration of interests

A.K. is a member of iScience’s Advisory Board and one of the Guest Editors of the Special Issue “Advanced thermal control: Fundamentals and applications.”

## Declaration of generative AI and AI-assisted technologies in the writing process

During the preparation of this work, the authors used Claude to improve the language and readability of the manuscript. After using this tool, the authors reviewed and edited the content as needed and take full responsibility for the content of the publication.

## STAR★Methods

### Key resources table

**Table:** 

REAGENT or RESOURCE	SOURCE	IDENTIFIER
**Software and algorithms**		

Ansys Maxwell 2024 R1 (magnetostatic and transient FEM simulations)	Ansys Inc.	https://www.ansys.com/products/electronics/ansys-maxwell
Ansys Twin Builder 2024 R1 (electrical circuit co-simulation)	Ansys Inc.	https://www.ansys.com/products/digital-twin/ansys-twin-builder

**Other**		

NdFeB N45 permanent magnet; Bakker Magnetics Neodymium Sintered Standard Grades Datasheet	Bakker Magnetics	https://bakkermagnetics.com/mta/permanent-magnets/neodymium-magnets/
Alnico 9 permanent magnet; Arnold Magnetic Technologies Alnico permanent magnets datasheet	Arnold Magnetic Technologies	https://www.arnoldmagnetics.com/products/alnico-magnets/
Ferrite Y30 permanent magnet; Eclipse Magnetics Ferrite/Ceramic Magnets Datasheet	Eclipse Magnetics	https://www.eclipsemagnetics.com/products/magnetic-materials-and-assemblies/magnet-materials/ferrite-ceramic-magnet-material/
M270-35A non-grain-oriented electrical steel (yoke); Isovac 270-35A datasheet	voestalpine Stahl GmbH	https://eva-steel.voestalpine.com/app/va-pds/range-of-supply/isovac_270-35_A_EF_EBD_K
Gadolinium (Gd) magnetic permeability data as a function of temperature and field strength (0°C–30°C); B-H curves derived from these data	Bouchekara et al.^62^	https://doi.org/10.2528/PIERM11062708

### Experimental model and study participant details

This study is computational and did not involve experimental models, biological samples, or study participants.

### Method details

#### Simulation software and tools

The active thermomagnetic generator (TMG) based on an electro-permanent magnet field source was analyzed numerically using the finite element method (FEM). Simulations were performed in Ansys Maxwell (2024 R1) using both magnetostatic (steady-state) and transient solution types. The overall simulation workflow is summarized in the flowchart (Figure S4). The numerical model couples Ansys Maxwell for geometric modeling and magnetic field simulations with Twin Builder for electrical parameters calculations. Magnetostatic post-processing was performed using Fields Calculator, while dynamic performance was evaluated through transient co-simulations.

The numerical methodology employed here has been previously validated against experimental measurements in our earlier works.^56^^,^^61^ In Tomc et al.,^56^ the same electro-permanent magnet-based magnetic field source that serves as the reference design in the present work was constructed as a physical prototype and experimentally characterized. Measurements of the magnetic flux density in the air gap, electric current, voltage, and temperature evolution were compared with FEM simulations performed in the same Ansys Maxwell/Twin Builder framework. As reported in,^56^ the numerically and experimentally determined energy efficiencies of the magnetic field source differed on average by 5.4%. The present work applies the same FEM framework to the parametric analysis of TMG performance. While the FEM methodology has been experimentally validated for the magnetic field source in,^56^^,^^61^ experimental characterization of the complete TMG system, including the Gd working material and electrical output, has not been performed in the present work and is identified as a direction for future experimental studies.

#### The reference magnetic field source

The electro-permanent magnet-based magnetic field source, originally developed and experimentally validated for magnetocaloric applications,^56^ serves as the reference (baseline) design for this study. The magnetic field source configuration, including the yoke, PM, and windings is shown in Figure 1. In the original design, the air gap was minimized to the value compatible with accommodating the Gd plate stack and fluid-flow channels, maximizing the flux density in the regenerator. This 4 mm gap is adopted as the baseline, anchoring the parametric study to a validated configuration.

The yoke is modeled as Isovac 270-35A alloy (total mass 26.8 kg); its magnetic and mechanical properties were taken from the specifications.^63^ The permanent magnet is neodymium-iron-boron NdFeB (Bakker Magnetics, N45-grade); its properties were taken from.^58^ To evaluate the influence of PM material, Alnico and hard ferrite were investigated as alternatives. Their key magnetic properties are summarized in Table 1.

The windings are modeled as copper. The TMG comprises layered gadolinium (Gd) plates (see inset in Figure 1A). The total mass of Gd working material in the reference configuration is 0.095 kg.

Temperature-dependent *B*-*H* curves (left inset of Figure 1A), which define the magnetization characteristics of Gd, were derived from the magnetic permeability data reported in ref.^62^ and cover the 0°C–30°C range, as summarized in Table S1. These curves were implemented in Ansys Maxwell to account for the nonlinear variation of relative magnetic permeability (*μ*_r_) throughout the thermal cycle.

#### Magnetic field source design variations

The reference design uses a NdFeB PM (18 mm thickness) and an air gap of 4 mm.^56^ This air gap is inherited from the original field source, where it was minimized while still accommodating the Gd plate stack and the fluid-flow channels, so as to maximize the flux density in the working material for the magnetocaloric application; it is adopted here as the baseline rather than re-optimized. To evaluate the influence of individual design parameters, each was varied sequentially relative to this baseline. Parametric magnetostatic and transient analyses were performed for the reference design and all configurations listed in Figure 2.

#### Simulation procedures and boundary conditions

In the model, the Neumann boundary condition was used at the outer region boundary (the external limits of the computational model), in which the magnetic field strength vector ($\vec{H}$) is tangential on these boundaries. At the internal interfaces between different objects within the region (for example, between Gd plates and the air gap), the natural boundary condition was used, in which the normal component of the magnetic flux density vector ($\vec{B}$) is continuous over the interface.

Adaptive mesh refinement was used to ensure high accuracy and solution stability. The desired precision for the magnetic field computation was defined within Ansys Maxwell by setting a maximum energy error of 1%. The mesh was automatically refined through iterative passes (with a maximum of 10 passes) until this specified convergence criterion was met before the main simulation process started. For each design configuration, both magnetostatic and transient simulations were performed, as illustrated in the workflow diagram (Figure S4).

Magnetostatic simulations were performed for the state in which one Gd plate assembly is at *T*_cold_ and the other at *T*_hot_; all simulation conditions are given in Figure 3. The resulting magnetic flux change Δ*Φ* between the two sides was computed as an estimate of the maximum achievable magnetic flux variation (Table 2).

Transient co-simulations were performed to model dynamic TMG operation, in which the two Gd plate assemblies oscillate in antiphase between *T*_cold_ and *T*_hot_ as a sinusoidal function of time, representing the TMG operating cycle. The simulation conditions and electrical circuit parameters are given in Figure 3; a load resistance of 50 Ω was connected in series with all four windings (electrical circuit schematic is provided in Figure S3). The load resistance of 50 Ω was selected based on preliminary transient simulations as the value at which maximum output voltage is achieved with minimal current drop for the reference geometry; the number of winding turns (750 per winding) was subsequently optimized through transient simulations to maximize the output power. The optimization results are provided in the supplemental information (Figure S2). Output parameters, their definitions and evaluation methods are listed in Table 2.

The principal model approximations, namely the neglect of magnetic hysteresis and eddy-current losses together with the assumption of a uniform working-material temperature, have only a minor effect on the predicted magnetic flux at the operating frequency of 1 Hz. Eddy currents are negligible: in all permanent magnets studied the electromagnetic skin depth at 1 Hz greatly exceeds the magnet dimensions, so the field penetrates fully, while in the yoke the laminated non-grain-oriented electrical steel (0.35 mm sheets^63^) suppresses induced currents. Hysteresis is not modeled explicitly, but the soft-magnetic yoke steel has a narrow *B*–*H* loop and low coercivity, so omitting the loop introduces only a small offset in the predicted flux, while the associated power dissipation scales with frequency and is correspondingly small at 1 Hz. During operation the permanent magnets are therefore subject only to these negligible eddy-current losses, and as only the Gd temperature varies in the model, the magnet temperature remains constant, so thermal demagnetization does not occur. The error from the uniform-temperature assumption was quantified by comparing uniform and linear-gradient Gd temperature profiles in magnetostatic simulations (Figure S1; Table S2), yielding a difference of less than 1.5% in the predicted magnetic flux change. These approximations therefore do not affect the conclusions of the parametric analysis.

The operating frequency *f* = 1 Hz was selected as a representative value typical for active TMG devices.^64^ The simulation duration of 5 s was selected to ensure that the output parameters reach a periodic steady state; only the last cycle (t = 4–5 s) was used for the analysis of output parameters. The temperature span of 0°C–30°C was selected to cover the magnetic phase transition of Gd (∼20°C), over which its magnetization changes significantly; this range is prescribed as an input parameter in the simulations, not derived from a thermal model. The present study focuses exclusively on the magnetic field source and does not include heat transfer modeling; the connection between the Gd operating temperature and the external heat source temperature (e.g., low-grade waste heat at 40°C–99°C) depends on the thermal management design of the complete TMG system and is beyond the scope of this work. For TMG applications targeting higher-temperature heat sources, working materials with correspondingly higher Curie temperatures would be employed; the magnetic field source optimization methodology presented here is applicable to such systems as well. In transient simulations, the temperature of each Gd plate assembly is assumed uniform along its length and varies sinusoidally in time between *T*_cold_ and *T*_hot_, with the two assemblies operating in antiphase—while one is at maximum temperature, the other is at minimum. A sinusoidal profile was chosen as a smooth, continuous function that ensures numerical stability and provides a consistent temperature input across all simulated configurations. The selection of these settings (Figure 3) does not affect the validity of the parametric analysis, since all configurations were simulated under identical conditions. The uniform temperature approximation adopted in the transient simulations has been validated against a linear temperature gradient scenario through magnetostatic calculations (supplemental information), yielding a difference of less than 1.5% in the predicted magnetic flux change. Since temperatures are prescribed as idealized simulation inputs rather than obtained from a coupled thermal analysis, the absolute values of the predicted electrical output represent performance estimates for the specified input conditions. The results of this parametric study are therefore best interpreted as comparative performance indicators, reflecting the relative differences between configurations rather than absolute output predictions for a specific operating system.

### Quantification and statistical analysis

No statistical analysis was performed. All results are based on deterministic finite element simulations.
