# Supplementary material for: Multiparametric evaluation of a magnetic field source for thermomagnetic generator
Source: iScience. 2026 Aug 27;29(9):117367. doi: 10.1016/j.isci.2026.117367 (PMC13546916; doi:10.1016/j.isci.2026.117367)
Supplement: Supplementary file 1 [file mmc1.pdf]

iScience, Volume *29*

Supplemental information

Multiparametric evaluation of a magnetic field source for thermomagnetic generator

Radel Gimaev, Andrej Kitanovski, and Katja Klinar

Supplemental Methods S1

Validation of the uniform temperature approximation

In real systems, the temperature distribution of the working material along the fluid flow is non-uniform, and a temperature gradient is present at any given time. Therefore, in this part of the study, the influence of the temperature gradient on the numerical simulation results was evaluated using magnetostatic calculations in Ansys Maxwell. The *B*-*H* curves of Gd used in all simulations are provided in Table S1.

Gd plates in the model were discretized into 15 equal layers along their length (and fluid flow). In the first scenario, a linear temperature gradient was applied: temperatures were set sequentially from 0 °C to 14 °C for the cold-side plates and from 16 °C to 30 °C for the hot-side plates, using a 1 °C step. A linear temperature gradient represents a reasonable first-order approximation of the thermal profile along the regenerator, where the temperature varies monotonically along the fluid flow direction. In the second scenario, a uniform average temperature approximation was used, with the plates set to 7 °C and 23 °C for the cold and hot sides, respectively. The uniform average temperature approximation was adopted to reduce computational complexity; its validity for the present geometry and operating conditions is verified by the comparative magnetostatic simulations presented in this section. The exact temperature assigned to each layer for both scenarios is given in Table S2; the corresponding temperature profiles are illustrated in Fig. S1.

Magnetostatic simulations were conducted for both scenarios using reference geometry to determine the magnetic flux at the coils locations.

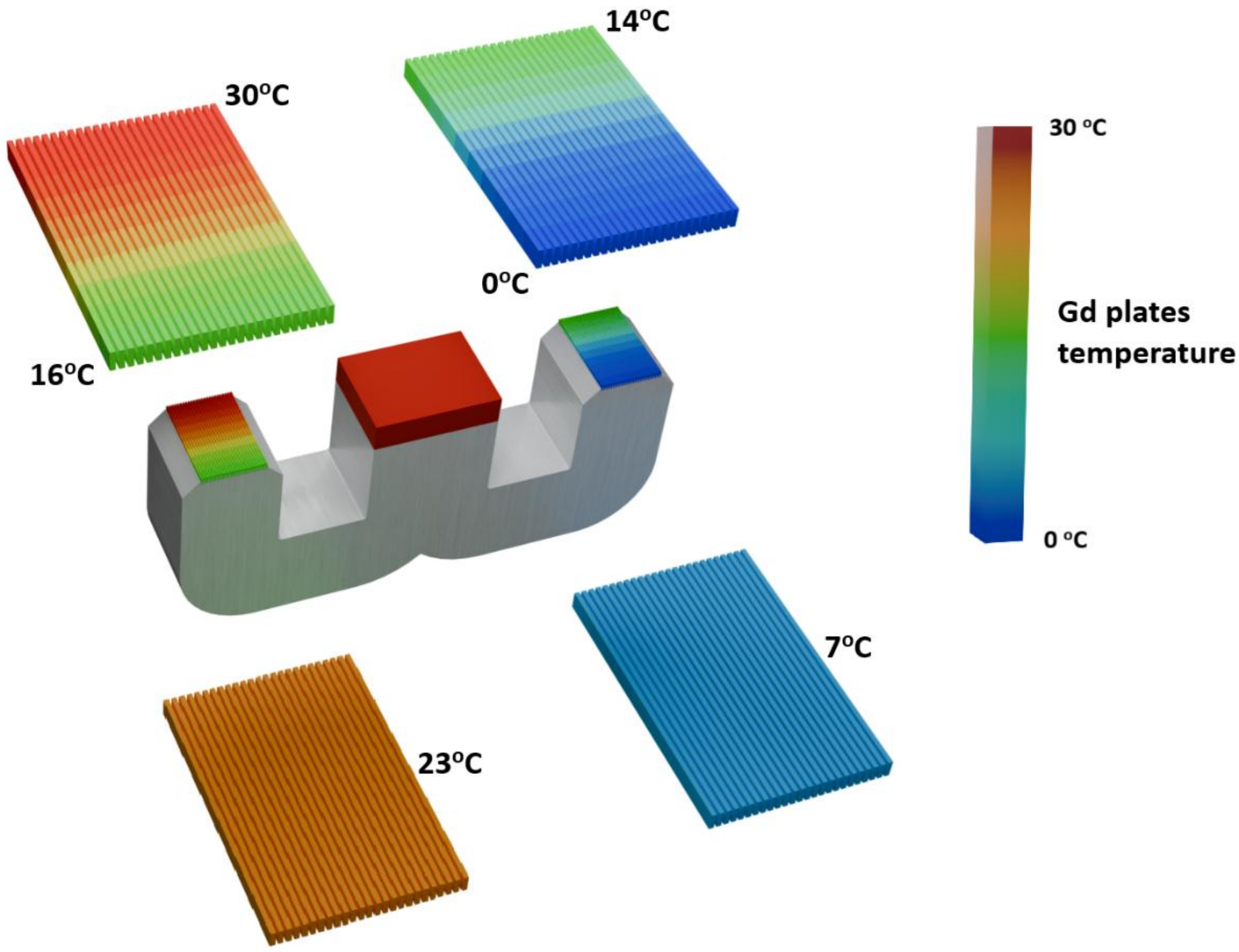

Fig. S1. Input temperature profiles for Gd plates in magnetostatic simulations, representing linear gradient (upper insets) and uniform (lower insets) thermal distributions.

Table S1. *B*-*H* curves of Gd at selected temperatures used in the magnetostatic simulations, derived from experimental magnetic permeability data [S1]

B (T)	H (kA/m)			
	0 °C	10 °C	20 °C	30 °C
**0**	0	0	0	0
**0.01**	0.3	0.4	1.1	3.1
**0.1**	4.2	5.8	13.1	35.1
**0.2**	12.9	19.4	40.5	73.9
**0.3**	33.8	46.9	76.2	113.5
**0.4**	63.4	81.9	122.0	148.3
**0.5**	98.5	122.6	159.4	198.6
**0.6**	142.5	167.7	206.9	242.6
**0.7**	181.3	212.4	262.5	296.8
**1**	298.1	347.7	411.6	459.8

Table S2. Temperature distribution along the Gd regenerator length for the linear gradient and uniform approximation scenarios, where *x* is the coordinate along regenerator length, measured from the cold fluid inlet.

x (mm)	Temperature distribution T (°C)			
	Hot-side regenerator		Cold-side regenerator	
	Linear	Uniform	Linear	Uniform
**4**	16		0	
**8**	17		1	
**12**	18		2	
**16**	19		3	
**20**	20		4	
**24**	21		5	
**28**	22		6	
**32**	23	23	7	7
**36**	24		8	
**40**	25		9	
**44**	26		10	
**48**	27		11	
**52**	28		12	
**56**	29		13	
**60**	30		14	

Note: Uniform: single temperature applied to all 15 layers.

A comparative analysis yielded a magnetic flux change $\Delta\Phi$ of 2.92×10^{-4} Wb for the linear gradient scenario and 2.88×10^{-4} Wb for the uniform approximation. The difference

between the two approaches is less than 1.5 %, validating the use of the uniform temperature approximation for all subsequent simulations without compromising accuracy.

Coil parameters optimization

The magnetostatic analysis determines the spatial distribution of magnetic flux independently of the electrical circuit parameters, as it solves only the static field equations without considering induced voltages or currents. Therefore, the number of coil turns does not affect the magnetostatic flux distribution but determines the induced voltage according to Faraday's law. Windings optimization was therefore performed using transient simulations.

In the original field source for the magnetocaloric device [S2], the windings were used to create a magnetic field with a current of 3.75 A through each winding. In the considered TMG, by contrast, the windings function as induction coils – the time-varying magnetic flux induces an electromotive force. As a result, the operating currents are two orders of magnitude lower than in excitation mode, which fundamentally changes the optimal winding parameters: a higher number of turns and thinner wire are preferable to maximize the induced voltage.

According to Faraday's law:

$$U = -N\frac{d\Phi}{dt},$$

where Φ is the magnetic flux through one turn and $d\Phi/dt$ is the rate of change of magnetic flux with time, an increase in the number of turns N in the coil contributes to an increase in the output voltage U. However, at the same time, the length of the wire increases and its cross-section decreases (with fixed coil dimensions), which leads to an increase in the internal resistance of the winding. This, in turn, can cause a reduction in the voltage on the load due to losses in the wire.

To determine the optimal number of turns, a series of transient simulations was conducted. Fig. S2 shows the dependence of the output power on the number of turns per winding at a load resistance of 50 Ohm. A load resistance of 50 Ohm was selected based on preliminary simulations as the value at which maximum output voltage is achieved with minimal current drop for the geometry and configuration considered in this study.

As a result of the simulation, it was determined that the optimal number is 750 turns for each of the four windings in this TMG design. This value was used in all subsequent transient simulations presented in this work.

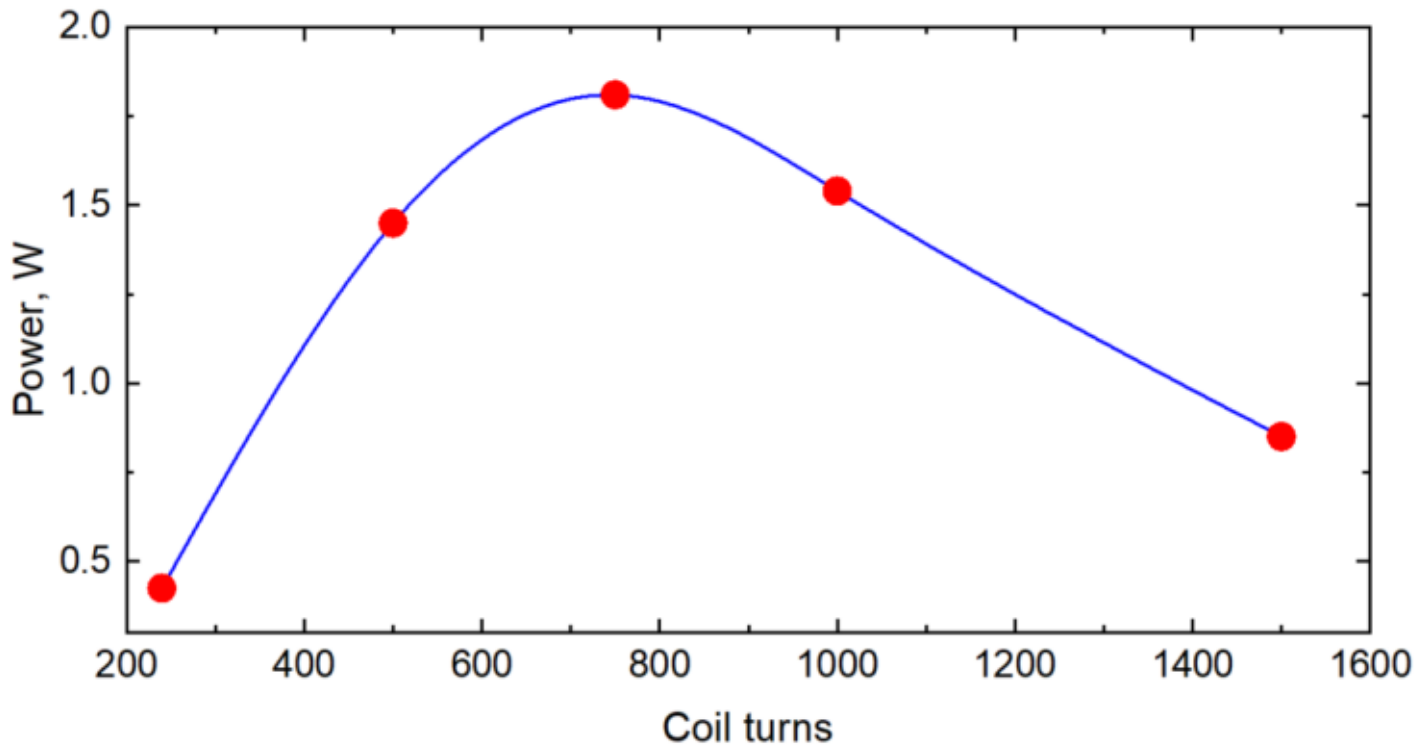

Fig. S2. Effect of the number of turns per one winding on the output power at a 50 Ohm load.

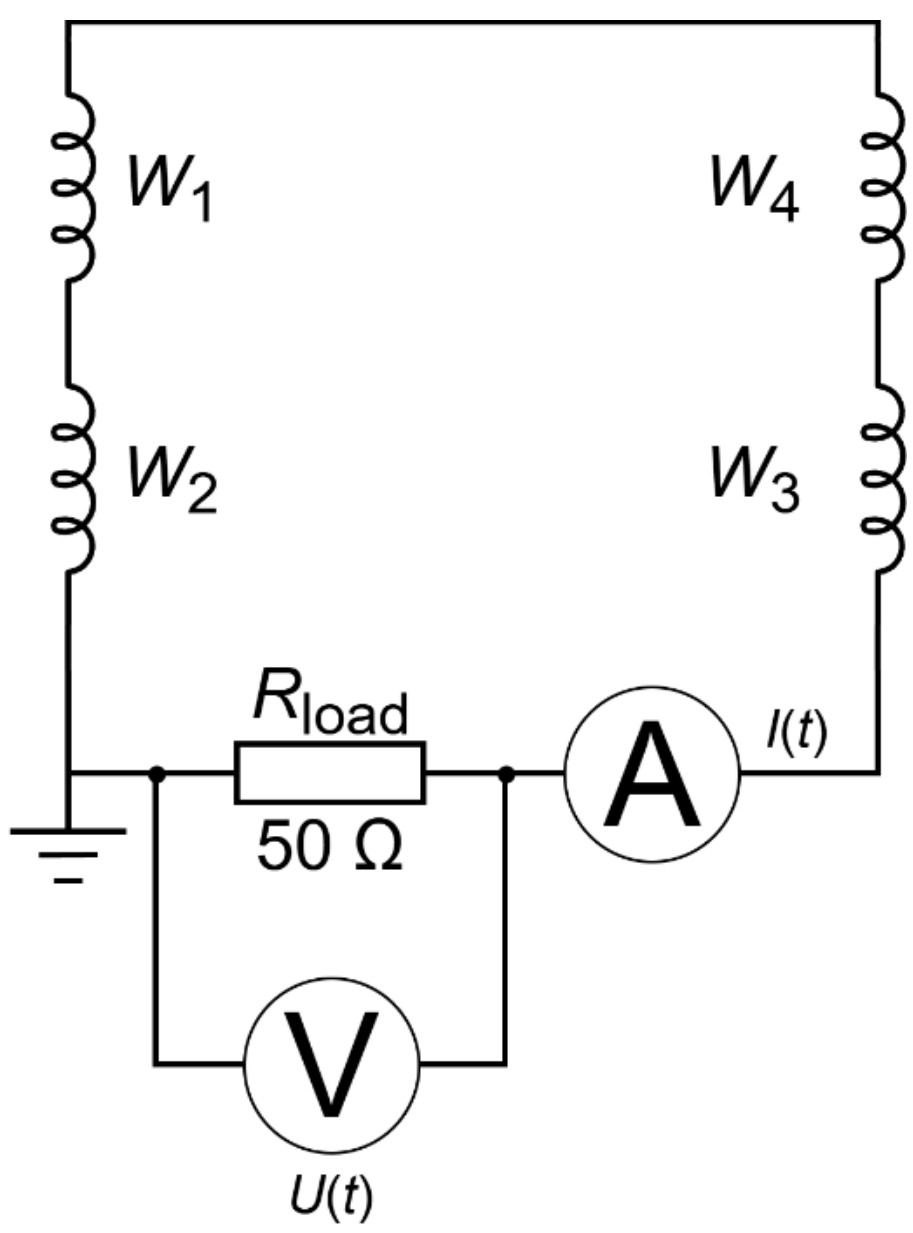

Fig. S3. Electrical circuit schematic used in transient co-simulations. Four windings (W_1–W_4) are connected in series with an amperemeter A and a load resistance R_{load} = 50 Ω. A voltmeter V is connected in parallel with R_{load}. $I(t)$ and $U(t)$ denote the current through the circuit and the voltage across the load, respectively.

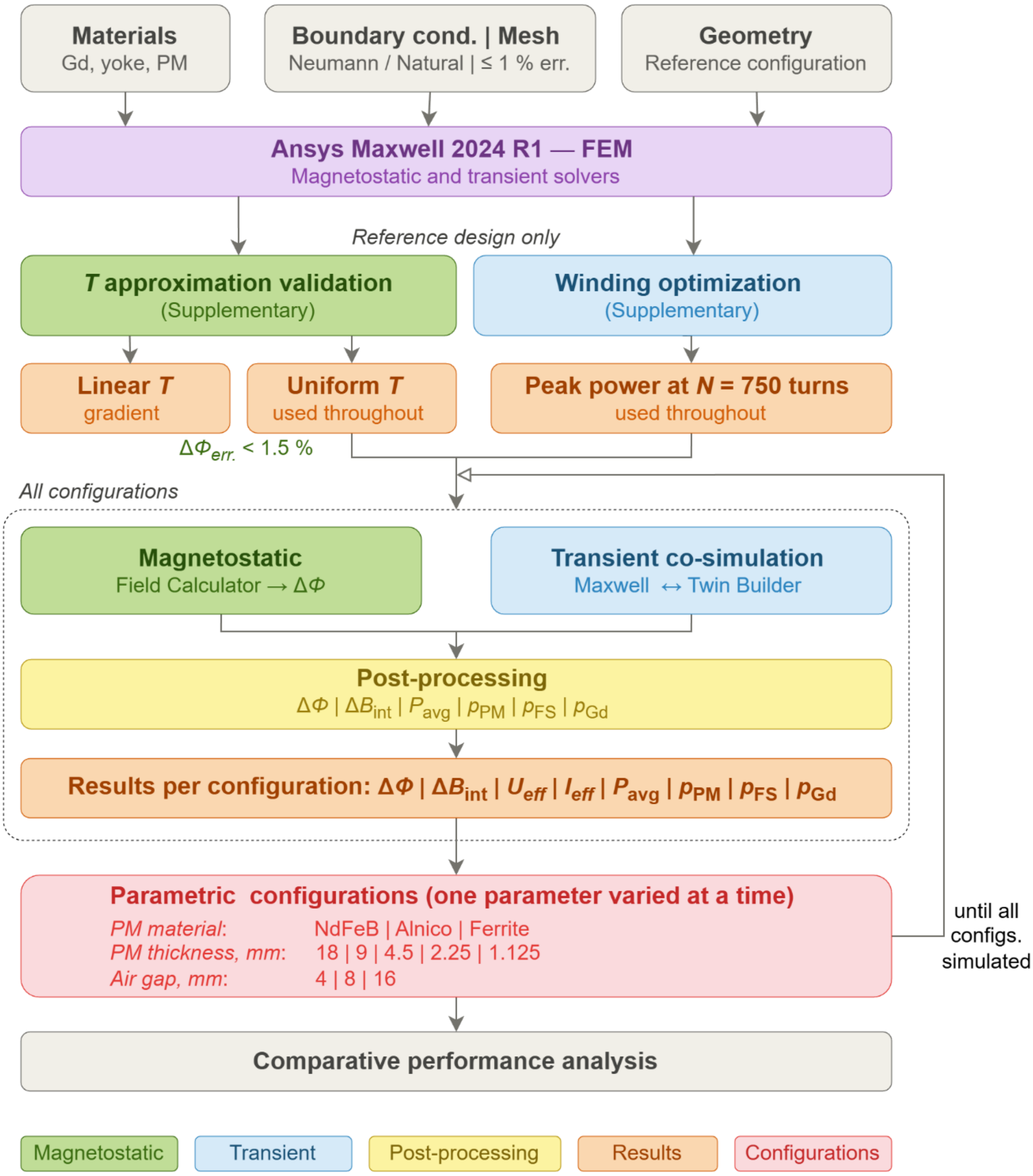

Fig. S4. Workflow of the numerical simulation, where green blocks denote magnetostatic simulations, blue - transient co-simulations, yellow - post-processing, orange - results and red - parametric configurations. Simulation inputs include material properties (working material gadolinium (Gd), yoke, permanent magnet (PM) and winding material), mesh settings, boundary conditions and geometry.

Magnetostatic simulations results

The complete magnetostatic simulation results for all parametric configurations are provided below. Conditions are given in Table 2 of the main text.

Table S3. Magnetostatic simulations results.

Magnet type	Magnet thickness (mm)	Air gap (mm)	Mass (kg)			ΔB_{int} (T)	$\Delta \Phi$ (mWb)	$\Delta \Phi$ per total mass (mWb/kg)
			Magnet	Yoke	Gd			
**NdFeB***	18	4	0.63	26.8	0.095	0.33	0.52	0.0189
**Alnico 9**	18	4	0.63	26.8	0.095	0.30	0.45	0.0163
**Ferrite**	18	4	0.41	26.8	0.095	0.25	0.37	0.0136
**NdFeB**	9	4	0.31	27.1	0.095	0.34	0.51	0.0185
**NdFeB**	4.5	4	0.16	27.3	0.095	0.30	0.47	0.0171
**NdFeB**	2.25	4	0.08	27.4	0.095	0.26	0.39	0.0141
**NdFeB**	1.125	4	0.04	27.4	0.095	0.24	0.36	0.0131
**NdFeB**	18	8	0.63	26.6	0.19	0.28	0.42	0.0153
**NdFeB**	18	16	0.63	26.3	0.38	0.24	0.35	0.0128

** Reference magnetic field source design*

Transient simulations results

The complete transient simulation results for all parametric configurations are provided below. Conditions are given in Fig. 4 of the main text.

Table S4. Effect of permanent magnet type on TMG output parameters and performance.

Magnet type	U_{eff} (V)	I_{eff} (A)	P_{avg} (W)	p_{PM} (W/kg)	p_{FS} (W/kg)	p_{Gd} (W/kg)
**NdFeB***	6.6	0.13	0.86	1.37	0.031	9.05
**Alnico 9**	5.2	0.11	0.55	0.87	0.02	5.79
**Ferrite**	2	0.04	0.09	0.22	0.003	0.95

** Reference magnetic field source design*

Table S5. Effect of reducing the thickness of the permanent magnet, obtained from transient simulations (conditions are given is Table 2 of the main text).

Magnet thickness (mm)	U_{eff} (V)	I_{eff} (A)	P_{avg} (W)	p_{PM} (W/kg)	p_{FS} (W/kg)	p_{Gd} (W/kg)
**18***	6.6	0.13	0.86	1.37	0.031	9.05
**9**	5.5	0.11	0.66	2.12	0.024	6.95
**4.5**	5.4	0.11	0.59	3.69	0.021	6.21
**2.25**	4.7	0.09	0.44	5.5	0.016	4.63
**1.125**	3.7	0.07	0.27	6.75	0.01	2.84

* *Reference magnetic field source design*

Table S6. Effect of increasing the air gap.

Air gap (mm)	U_{eff} (V)	I_{eff} (A)	P_{avg} (W)	p_{PM} (W/kg)	p_{FS} (W/kg)	p_{Gd} (W/kg)
**4***	6.6	0.13	0.86	1.37	0.031	9.05
**8**	6.8	0.14	0.92	1.46	0.034	4.84
**16**	6	0.12	0.73	1.16	0.027	1.92

* *Reference magnetic field source design*

REFERENCES

[S1] H.R.E.H. Bouchekara, A. Kedous-Lebouc, J.P. Yonnet, ELECTROMAGNETIC DESIGN OF A MAGNETIC FIELD SOURCE FOR A MAGNETOCALORIC REFRIGERATOR, Prog. Electromagn. Res. M 19 (2011) 251–263. https://doi.org/10.2528/PIERM11062708.

[S2] U. Tomc, S. Nosan, K. Klinar, A. Kitanovski, Towards powerful magnetocaloric devices with static electro-permanent magnets, J. Adv. Res. 45 (2023) 157–181. https://doi.org/10.1016/j.jare.2022.05.001.
